# Exonization of the LTR transposable elements in human genome

**DOI:** 10.1186/1471-2164-8-291

**Published:** 2007-08-28

**Authors:** Jittima Piriyapongsa, Nalini Polavarapu, Mark Borodovsky, John McDonald

**Affiliations:** 1 School of Biology, Georgia Institute of Technology, Atlanta, Georgia 30332, USA.; 2 Wallace H. Coulter Department of Biomedical Engineering, Georgia Institute of Technology and Emory University, Atlanta, Georgia 30332, USA.; 3 Division of Computational Science and Engineering at College of Computing, Georgia Institute of Technology, Atlanta, Georgia 30332, USA.

## Abstract

**Background:**

Retrotransposons have been shown to contribute to evolution of both structure and regulation of protein coding genes. It has been postulated that the primary mechanism by which retrotransposons contribute to structural gene evolution is through insertion into an intron or a gene flanking region, and subsequent incorporation into an exon.

**Results:**

We found that Long Terminal Repeat (LTR) retrotransposons are associated with 1,057 human genes (5.8%). In 256 cases LTR retrotransposons were observed in protein-coding regions, while 50 distinct protein coding exons in 45 genes were comprised exclusively of LTR RetroTransposon Sequence (LRTS). We go on to reconstruct the evolutionary history of an alternatively spliced exon of the Interleukin 22 receptor, alpha 2 gene (*IL22RA2*) derived from a sequence of retrotransposon of the Mammalian apparent LTR retrotransposons (MaLR) family. Sequencing and analysis of the homologous regions of genomes of several primates indicate that the LTR retrotransposon was inserted into the *IL22RA2 *gene at least prior to the divergence of Apes and Old World monkeys from a common ancestor (~25 MYA). We hypothesize that the recruitment of the part of LTR as a novel exon in great ape species occurred prior to the divergence of orangutans and humans from a common ancestor (~14 MYA) as a result of a single mutation in the proto-splice site.

**Conclusion:**

Our analysis of LRTS exonization events has shown that the patterns of LRTS distribution in human exons support the hypothesis that LRTS played a significant role in human gene evolution by providing cis-regulatory sequences; direct incorporation of LTR sequences into protein coding regions was observed less frequently. Combination of computational and experimental approaches used for tracing the history of the LTR exonization process of *IL22RA2 *gene presents a promising strategy that could facilitate further studies of transposon initiated gene evolution.

## Background

Retrotransposon sequences comprise more than 40 % of the human genome [1,2]. Once dismissed as "junk DNA" of little or no adaptive significance [3,4], retrotransposons and other classes of transposable elements (TEs) are now generally considered as significant contributors to gene and genome evolution [5-9]. Of particular interest has been the ability of TEs to contribute to exon evolution by "exonization", i.e., an insertion of a TE into an intron and subsequent recruitment of this sequence or its part into a new protein-coding exon [10]. For example, it has been estimated that 5% of all alternatively spliced human exons had been derived from the exonization of Alu elements [11-13].

LTR transposable elements comprise nearly one-tenth of the human genome and have been implicated in the cis-regulatory evolution of a number of human genes [5,6,14-18]. The structure of a complete LTR retrotransposon (autonomous mobile element) comprises two copies of long terminal directed repeats (LTRs) flanking an internal region containing *gag *and *pol *genes, which encode a protease, reverse transcriptase, RNase H and integrase. These protein products are necessary for the formation of virus-like particles (VLPs) wherein replication of the element takes place. Some elements evolved from retroviruses have additional open reading frames (ORFs), e.g. *env *gene [1,19]. Flanking LTRs contain all the necessary transcriptional regulatory elements.

Although global database screens have been conducted to examine the contribution of TEs to human protein-coding regions [10,20,21], none have concentrated specifically on the prevalence of the LRTS-derived protein-coding exons of human genes. Here we report the results of computational analysis of the LRTS exonization in human genome. Also we describe the plausible scenario of the exonization process of an alternatively spliced exon of the alpha 2 gene of the Interleukin 22 receptor (*IL22RA2*) supported by new experimental data.

## Results and Discussion

### Updated list of LRTS-associated genes

To identify incidences of LRTS exonization, the annotation of human exons given in the UCSC genome browser was compared with the annotation of transposable elements available in the same source. We detected LRTS associations in 1,057 out of 18,241 genes (5.8 %). These associations include 1,249 distinct exons participating in 1,287 transcripts (note that a particular exon is counted once though it may participate in several alternative transcripts). It was reported earlier [10] that 130 out of 13,799 human genes (0.9 %) were found to contain LRTS in protein coding regions. In comparison, in our data set (18,241 genes/23,821 transcripts) we observed LRTS associations with protein-coding exons in 256 genes (1.4 %). Current LRTS search done at the DNA instead of mRNA level helped detect several short LRTS-exon overlaps that could be missed at mRNA level. Interestingly, only 53 of the previously reported 130 cases were found in current analysis using the updated RefSeq gene data. Many previously identified cases (61 cases) did not show up in our data set as the earlier sequences were removed, suppressed, or replaced. Several cases appear to be possible false positives. In one case, LRTS was detected in UTR instead of in CDS. No LRTS was detected in other two cases when the RepeatMasker program was run separately on each mRNA sequence using its specific G+C content, which gives a slightly more accurate result, as opposed to input of multiple sequences with averaged G+C content used in the program [22].

### Distribution of LRTS in human exons

We found that human gene exons (either protein-coding or non-coding) overlap with LTR flanks of LTR elements more frequently (1,074 cases) than with internal sequences (242 cases; note that exons overlaped with both regions were counted twice). This observation could be related to the fact that most (85%) of the LTR retroposon-derived sequences in human genome consist only of a solo LTR, with the internal sequence lost due to homologous recombination between the flanking LTRs [1]. Upon checking by BLASTX of 242 exons overlapping with the internal sequences, 61 exons were found to contain a section or even a whole viral gene (*i.e. gag*, *pol*, and *env*). However, only 20 of these 61 exons were protein-coding exons. Moreover, only in 10 cases was the reading frame of a human gene the same as the one of the viral gene. Seven out of these ten cases were observed in hypothetical genes. The remaining three cases represented a gene for endogenous retroviral protein, syncytin (*ERVWE1*), a gene for Krueppel-related zinc finger protein(*H-plk*) and a placenta-specific gene (*PLAC4*) which protein products contain the envelope, envelope and gag viral protein domain, respectively. All three genes are preferentially expressed in the placenta [23-25]. This observation indicates that the invasion of the Human Endogenous Retrovirus (HERV) may contribute to molecular mechanisms involved in human reproduction [26].

The majority of exons overlapping with LRTS (1,123 of 1,249) contain sequences homologous to only one LRTS. Exons overlapping with more than one LRTS were observed as well (Table 1). Overall, we have found 1,395 associations (overlaps) between an LRTS and an exonic sequence. These 1,395 observations were classified further according to the extent of LRTS overlap with an exon (Table 2), type of exon (Table 3), and LRTS class/family (Table 4). The majority of LRTS associations with genes (586/1395 or 42 %) constitute an apparent extension of original exon due to activation of alternative splice site located inside LRTS. On the other hand, in 22.9% (319/1395) of these associations LRTS was recruited as an entirely novel exon (Table 2).

**Table 1:** The distribution of the number of LTR elements (either partial or full elements) containing in an exon

**number of LTR elements overlaps with an exon**	**number of exons**	**number of associations**
1	1123	1123
2	108	216
3	16	48
4	2	8

Total	1249	1395

**Table 2:** The distribution of the extent of overlap between an exon and an LTR element

**extent of LRTS overlap**	**number of associations**
An LRTS completely covers an exon	319
An LRTS partially overlaps (5' or 3' boundaries) with an exon	586
An LRTS is situated within an exon	490

Total	1395

**Table 3:** The distribution of type of exons containing LRTS

**exon type**	**number of associations**
5'UTR exon	245
3'UTR exon	196
first CDS exon	127 (41 in the 5'UTR, 8 in the CDS region and 78 span both regions)
last CDS exon	571 (484 in the 3'UTR, 16 in the CDS region and 71 span both regions)
single protein coding exon	152 (17 in the 5'UTR, 97 in the 3'UTR, 11 in the CDS region and 27 span both UTR and CDS regions)
internal protein coding exon	72
more than one type of exon (for a particular exonic sequence)	32

Total	1395

**Table 4:** The distribution of class/family of LRTS containing in an exon

**LRTS Class/family**	**number of associations**
ERV1	513
ERVK	58
ERVL	249
MaLR	575

Total	1395

Regarding the distribution of LRTS within a complete gene structure (5'UTR, first CDS exon, internal protein coding exons, last CDS exon, 3'UTR), the LRTS fragments were found in untranslated regions (UTRs), mainly in 3'UTRs, much more frequently than in protein-coding (CDS) regions. This observation is consistent with the previous study [27] and indicates the putative role of LRTS in resident gene regulation by providing sequence material for emerging regulatory sequences [6,17]. In comparison, insertion of LRTS in a protein coding region may interfere with gene function, and in many cases such a modification is likely to be eliminated by negative selection. Note that an LRTS was found more frequently in the last CDS exon, especially in the exon untranslated region, and less frequently in internal coding exons (Table 3).

### LRTS-derived protein coding exons

We have found 50 protein coding exons completely derived from LRTS (41 internal, 2 initial, 4 terminal coding exons and 3 single coding exons, see additional file 1: suppl_table_1.pdf for details). Most of LRTS-derived exons (36/50) were comprised exclusively of LTR flanking regions. Eleven exons were derived from LTR element internal sequences and 3 exons contained both types of regions. Of the 50 exons, 38 were components of well characterized protein coding genes (i.e., genes with the corresponding mRNAs available in GenBank and with encoded proteins listed in SWISS-PROT, TrEMBL, and TrEMBL-NEW).

The low frequency of protein coding exons fully derived from LRTS indicates that the chance of a successful recruitment of a whole coding exon from the LTR transposable element is rather small. The exonization of originally intronic LRTS requires the presence of a pair of potential splice sites, enclosing a sequence with no stop codon in the appropriate reading frame. Also, the amino acids contributed by a mobile element should not disrupt the structure of a protein encoded by the original gene, particularly, the addition of a new exon should not change the coding frame for the remaining part of a gene.

Interestingly, most of the protein coding exons derived entirely from the LTR flanking regions originated from the MaLR family (24 out of 36). This could be explained by several factors. First of all, MaLR elements make up about 50% of the LTR retroelements in the human genome [1], and this high frequency alone may relate to their overrepresentation in protein coding exons. MaLRs are also relatively ancient elements, which have probably been exposed to more opportunities for exonizations over time. Note that the age factor has been implicated for proliferation of Alu-derived exons as well [12]. Finally, it is a formal possibility that nucleotide sequences of the MaLR family are better amenable for derivation of protein coding exons.

The internal sequence of MaLR is rarely found retained in the human genomic sequence [28]. Particularly, among exons derived from the internal parts of LRTS only one was from the MaLR family.

### Contribution of LRTS to gene transcripts

We further analyzed the abundance of LRTS-derived exons in gene transcripts. Most of the 275 genes containing at least one exon completely derived from LRTS (201 out of 275) are single transcript genes while the remaining 74 generate more than one transcript per gene. Note that about 60% (121/201) of single transcript genes encode zinc finger proteins (25%) or hypothetical proteins (35%). Apparently for the single transcript gene the LRTS insertion either has not disrupted the host gene function or possibly provided some beneficial modulation of the initial function and thus has been tolerated by natural selection.

In 55 out of 74 genes (74.3%) with multiple transcripts, LRTS-derived exons were present in some transcript variants, but not in all of them. This observation corresponds to the scenario whereby recruiting of LRTS into alternatively spliced exon allows the main transcript to maintain the function while the LRTS-associated exons are "examined" by natural selection, which may lead to emergence of transcripts with new functions.

We also found that most of the LRTS-derived protein coding exons (48/50) were either alternatively spliced ones or the components of single transcript genes. In contrast, most of LRTS derived constitutive exons (those that are present in all alternative transcripts) are found in 5'UTR sequences. This observation indicates that novel cis-regulatory sequences supplied by LTR elements to human genes are more likely to be fixed in evolution than sequences supplying protein coding domain which are used as alternative ways to create protein variability.

### Reconstruction of evolution of *IL22RA2 *gene (transcript variant 1)

**Figure 1:**
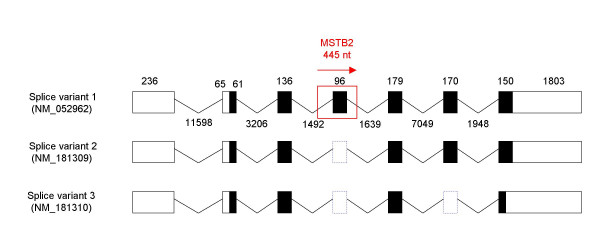
**Exon-intron organization of human *IL22RA2 *gene**. Exon and intron sequences are represented by boxes and angular lines, respectively, with lengths indicated in base pairs. Coding and untranslated regions are represented by filled boxes and open boxes, respectively while the blue dashed boxes demonstrate the absence of the exon sequences on mRNA level. The region showing homology with MSTB2 is labeled in red border. A horizontal arrow indicates the LTR orientation.

The *IL22RA2 *gene has an internal protein coding exon derived from an LTR flanking sequence. This gene encodes the only soluble receptor [29] in the class II cytokine receptor family (CRF2). IL22RA2 protein specifically binds to interleukin 22 (IL22) and by preventing the interaction of IL22 with its cell surface receptor, neutralizes IL22 activity [30-32]. Three alternatively spliced transcripts of the *IL22RA2 *human gene encoding three protein variants (263, 231 and 130 amino acids in length) have been described earlier [30]. The longest transcript (variant 1) is generated (Fig. 1) by addition of the 96 nt exon (exon 3/4) to splice variant 2 between exon 3 and exon 4 [30,31,33].

**Figure 2:**
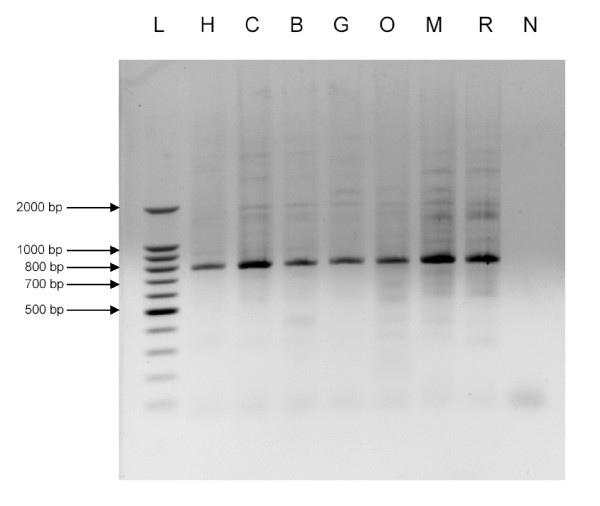
**PCR-sequencing**. Agarose gel electrophoresis of *IL22RA2 *homologous regions carrying LTR, MSTB2, from seven primates amplified by PCR. L, ladder; H, human; C, chimpanzee; B, bonobo; G, gorilla; O, orangutan; M, crab eating macaque; R, rhesus monkey; N, non-template control.

**Figure 3:**
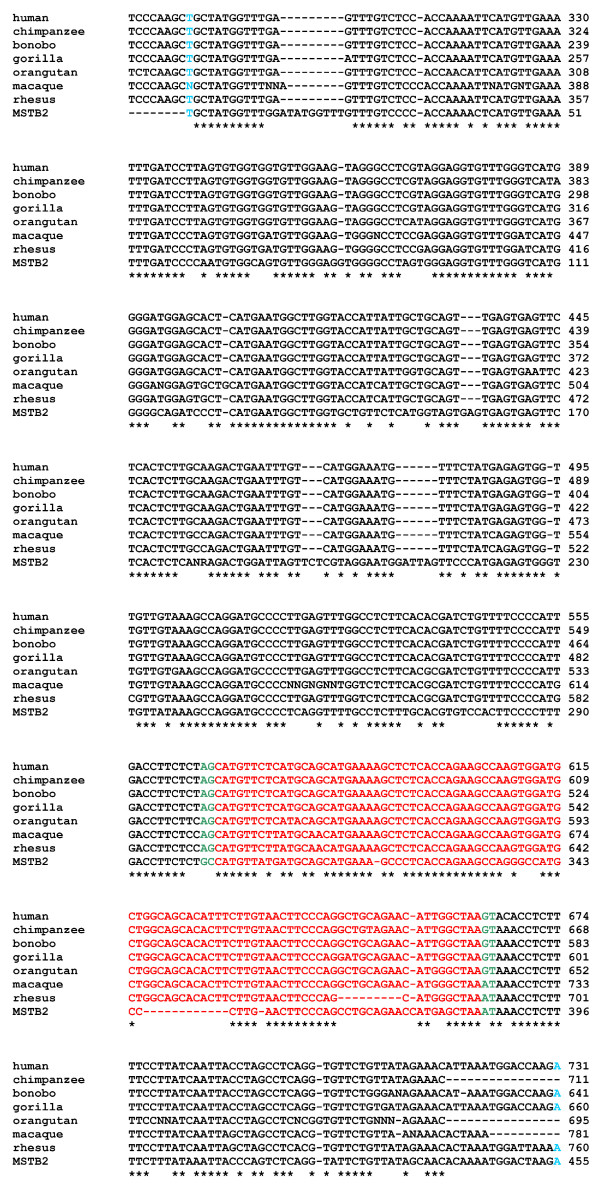
**Multiple sequence alignment of PCR products**. The PCR products are aligned and compared to the consensus sequence of MSTB2. The light blue letters indicate the start and end of LTR boundaries. The target exons and sequences in place of splice sites are shown in red and green color, respectively.

**Figure 4:**
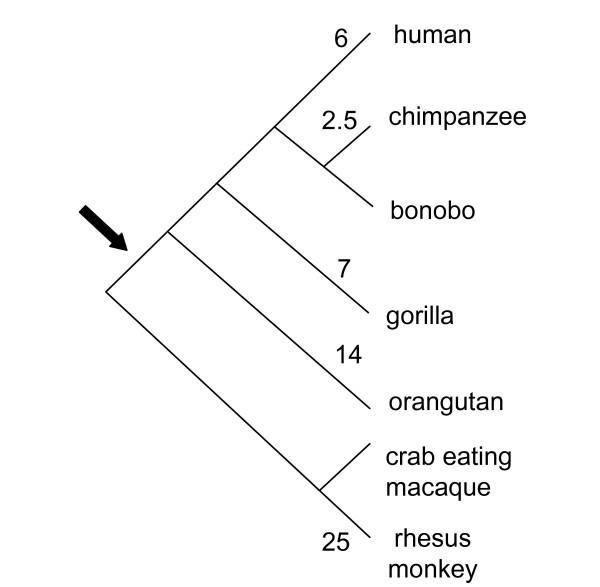
**Evolutionary history of *IL22RA2 *gene**. A phylogenetic diagram of seven primates selected in this study. The numbers next to branches on the tree show the approximated divergence time from the last common ancestor in million-year time units (MYA). The arrow indicates the estimated point of emergence of the target exon caused by the LTR mutation from AT to GT making the canonical donor site.

In the current study, we provide experimental data and computational analysis that show evolutionary evidence of exonization of LRTS invaded the human *IL22RA2 *gene. The exon 3/4 of the *IL22RA2 *gene (transcript variant 1) is situated within the LTR sequence of MSTB2 subfamily of MaLR family (found in the same orientation as the coding sequence (Fig. 1)). The sequence alignment of the particular LTR and the MSTB2 LTR consensus sequence shows 82.8 % identity (for ungapped part of the 431 nt long alignment). The exon 3/4 contributes 32 amino acids to the IL22RA2 protein product without changing reading frame for the rest of the protein. A homologous exon was not found either in the mouse or in the rat orthologous gene. Weiss et al. 2004 [29] also indicated that a counterpart of this exon was absent in mouse and rat. The functionality of the LTR exonization is corroborated by the existence of the mRNA sequences containing the exon 3/4 [RefSeq:NM_052962, GenBank:AY040567, AY358737, EMBL:AJ313162]. The data available at the UCSC genome browser show that the MSTB2 derived sequence is conserved in chimpanzee and rhesus monkey while is absent in other vertebrates. To extract the sequences homologous to the exon 3/4 in seven primates: human, chimpanzee, bonobo, gorilla, orangutan, crab-eating macaque and rhesus monkey, we have performed the PCRs with human DNA derived primers (see methods), which generated well interpretable PCR products for all species (Fig. 2). We used newly determined PCR product sequences as well as publicly available genomic sequences of human, chimpanzee and rhesus monkey to construct the multiple sequence alignment. We observed that the splice sites flanking the target exon in all species but the crab-eating macaque and the rhesus monkey followed the GT/AG rule. In the other two species, we observed AT instead of GT at the donor site (Fig. 3). Therefore, emergence of this exon was likely to occur in ape lineage earlier than the divergence of orangutans and humans (Fig. 4). This event was mediated by the single transition from A to G yielding canonical donor splice site consensus. Note that AT (or GT in other cases) is positioned in the predicted LTR polyadenylation site A**AT**AAA (Fig. 3). Contrary to the acceptor site, the strength of the donor site depends on the presence of just a few specific nucleotides around GT consensus. Therefore, a single mutation might create a functional donor splice site. The canonical dinucleotide (AG) of the acceptor site appeared in all primates we have studied. However, this dinucleotide is different from dinucleotide (GC) situated in the same position in MSTB2 consensus sequence (Fig. 3). One possibility is that the mutation of GC to AG could happen earlier in the primate lineage. However, the sequence logo generated from the multiple sequence alignment of the 880 MSTB2 sequences existing in the human genome shows low degree of conservation in the vicinity of acceptor site. Therefore, the dinucleotide predecessor of AG should not necessarily be the consensus GC dinucleotide.

Several coincidences must have been involved in creation of the exon 3/4. The viable structural elements of the splice sites (GT/AG) were created by mutations. With the upstream intron in phase 2, the exon 3/4 emerged in the frame which had no stop codons inside, while the other two possible phases of intron would cause premature termination of translation. The new exon 3/4 (with length divisible by three) did not disrupt the global reading frame and therefore did not change the downstream amino acid sequence known to be important for ligand binding [33]. Our findings show that the exon 3/4 of *IL22RA2 *might be active and be expressed in the Great Apes, while we have not confirmed its expression in the Old World monkeys. This observation indicates that the exon 3/4 is likely to possess functional properties and it is an alternatively spliced exon. We have evaluated the possibility that the exon 3/4 is the subject for positive selection by the standard test based on non-synonymous K_a _to synonymous K_s _divergence rates ratio. There are three nonsynonymous substitutions between human and orangutan homologous exonic sequences, while there are no synonymous substitutions. The use of the Laplace pseudocounts produces (K_a_+1)/(K_s_+1) > 1, which indicates possible positive selection.

To date, very little is known about the role and the origin of this additional exon (exon 3/4) in transcript variant 1. Being the only CRF2 protein with 32 amino acids inserted adjacent to the region important for ligand recognition, this isoform may bind to structurally different ligands than other isoforms [33]. This possibility is supported by the experimental data which show that this variant fails to block IL22 activity [31]. The longer MaLR-related isoform may also modulate tissue-specific expression. The available data show that the *IL22RA2 *isoform 1 is expressed only in placenta while isoform 2 is highly expressed in placenta and mammary gland and at a lower level in spleen, skin, thymus and stomach [33]. However, nothing is known about the factors that control the expression of this longest *IL22RA2 *variant. Additional experiments should be performed to determine its function as well as to identify the possible change in ligand specificity due to the LTR-derived protein modification.

## Conclusion

The distribution of LTR elements that became parts of human protein-coding genes shows the distinct preference of LRTS fixation in 5' and 3'untranslated regions. These observations confirm existing concept of LRTS role as a contributor to gene regulation evolution. On the other hand, the recruitment of LRTS to encode a part of a protein domain leading the exaptation to evolution of the host gene is a less frequent event. As shown in the part of this paper related to evolution of *IL22RA2 *gene, several coincidences are necessary to allow the LRTS exonization event. The evolutionary analysis elucidates the action of the mechanism of incorporation of LRTS into a novel alternatively spliced exon.

## Methods

### Bioinformatic analysis

The refGene file (hg17, May2004) with data on 18,241 RefSeq human genes (genes on chr_random excluded) including alternatively spliced variants (23,821 transcripts in total) was retrieved from the UCSC genome browser [34]. The annotations of 254,542 exons were compared with the transposable elements annotations available in the same database to determine the frequency of the LTR elements in the exon regions. The descriptions of the LTR elements were provided in the Repbase update (Repbase release 8.12) [35]. We detected exon overlaps with the LTR flanking regions and/or the internal sequences of LTR elements. The overlaps with exons were labeled as complete (LRTS covers the whole exon), partial (LRTS partially overlap with exon), or inside overlap (LRTS completely inside the exon). The type of exons associated with LRTS were then classified as the first CDS exon (first exon containing coding sequence), the last CDS exon (last exon containing coding sequence), single protein coding exon (exon containing the whole CDS of a gene), 5'UTR exon (exon located upstream to the first CDS exon/single protein coding exon) and 3'UTR exon (exon located downstream from the last CDS exon/single protein coding exon), and internal protein coding exon (all other CDS exons). In cases of the first and last CDS exons as well as single protein coding exons, LRTSs could be inserted in either the UTR and/or the CDS region. Finally, all the initial results (see additional file 2: suppl_table_2.xls) were further processed. Exons identical in different transcripts were clustered to remove the redundancy. The LRTS fragments were reconstructed manually based on the initial data (e.g., LRTS family, human genome coordinates) and the LRTS information available in Repbase [35].

For all genes containing LRTS-derived exons, we used data of the Entrez gene and UCSC genome browser to infer information on alternative transcripts containing LRTS derived exons. Additionally, we checked the consistency of the reading frames in the exon overlaps with internal sequences of LRTS. The internal sequences of the LTR elements overlapping with CDS regions were translated in six reading frames and searched by BLAST and Pfam for the presence of domains of common viral proteins (*gag*, *pro*, *RT*, *RNaseH*, *IN *and *env*). The cases when detected viral protein domains became parts of human proteins were registered.

Given that the first and last CDS exons are commonly less reliably identified than the internal coding exons, we have considered further only 41 internal coding exons completely covered with LRTS. We have chosen exon 3/4 of the *IL22RA2 *gene (splice variant 1) for in depth study using PCR-sequencing of homologous regions of several primate genomes and comparative analysis of the sequence data.

The primers flanking the target LTR-derived sequence were designed to be specific to the conserved human-chimpanzee-rhesus monkey region (data from the UCSC genome browser) using the PRIMER3 program [36].

Sequences of PCR fragments were aligned by the ClustalW program with default parameters [37] and then were manually adjusted. For human, chimpanzee, and rhesus monkey, the annotated sequences of regions in question were previously available. In these three cases the known annotated sequences were used in the alignment while the PCR data were utilized as a complementary information. The donor-acceptor sites of the target exon were marked for all sequences based on the corresponding positions in the human *IL22RA2*. The timing of the exonization event was estimated via the phylogenetic analysis.

### PCR amplification of the *IL22RA2 *target exon

The PCR amplifications of genomic DNA of seven primate species (*Homo sapiens, Pan troglodytes, Pan paniscus, Gorilla gorilla, Pongo pygmaeus, Macaca fascicularis, Macaca mulatta*) were carried out by using the following primers, a forward primer 5'-ACCGCTACGACTTCTCTCTAC-3' and a reverse primer 3'-TCAGGTATTCTGGGGTCTG-5', which yield a 792 bp amplicon covering the region of the LTR in human. The PCR cycle conditions were as follows: initial 4 min and 30 sec pre-denaturation at 94°C, 30 cycles of 30 sec denaturation at 94°C, 30 sec annealing at 50°C, 1 min elongation at 72°C, and a final 1-cycle extension of 7 min at 72°C. The PCR products were then purified on 1% (w/v) agarose gel, Gibco BRL Ultra-Pure, visualized by ethidium bromide staining and extracted by using the gel extraction kit (QIAGEN). Direct sequencing of the PCR products was performed by the DNA Sequencing Services, of the Genomics Core Facility at the Georgia Institute of Technology.

## Authors' contributions

JP performed the bioinformatics analysis, carried out the PCR analysis, participated in the design of the study and drafted the manuscript. NP helped to design and implement the experimental analysis. MB conceived the computational study, carried out major editorial work with the manuscript and gave the final approval of the version to be submitted. JM conceived the experimental study, contributed in its design and participated in editing the manuscript. All authors read and approved the final manuscript.

## Supplementary Material

Additional file 1 This file contains the details of 50 LRTS-derived protein coding exons. Click here for file

Additional file 2 This file contains the information of all human exons associated with LRTS. Click here for file
