# Supplementary material for: Exonization of the LTR transposable elements in human genome
Source: BMC Genomics. 2007 Aug 28;8:291. doi: 10.1186/1471-2164-8-291 (PMC2008291; doi:10.1186/1471-2164-8-291)
Supplement: Additional file 1 — This file contains the details of 50 LRTS-derived protein coding exons. [file 1471-2164-8-291-S1.pdf]

Supplementary Table 1: Features of LRTS-derived protein coding exons.

	RefSeq gene[1]	strand (gene/ LRTS)[2]	exon no/ total exon[3]	exon length, nt[4]	protein length, aa	LRTS length, nt[5]	LRTS sub-family	LRTS class/ family[6]	Gene annotation	GO descriptions[7]	GenBank mRNAs with exonized LRTS[8]	ESTs with exonized LRTS[9]	sequences in place of splice sites flanking orthologous exons[10]
**internal protein coding exon**													
1	NM_005799*	+/-	34/36*	90(30)	1582	368	MSTD	MaLR[1]	InaD-like protein (*INADL*), transcript variant 3	protein binding (protein-protein interaction mediated by PDZ domains)	AJ224748	N/A	* GT/AG
2	NM_198712*	-/-	4/6*	70	400	265	MSTA	MaLR[1]	prostaglandin E receptor 3 (subtype EP3) (*PTGER3*), transcript variant 2	prostaglandin E receptor activity, ligand-dependent nuclear receptor activity, rhodopsin-like receptor activity	D86097	N/A	* donor (all = AT); acceptor (c & h = AG, r = TG)
3	NM_198713*	-/-	3/5*	76	393	265	MSTA	MaLR[1]	prostaglandin E receptor 3 (subtype EP3) (*PTGER3*), transcript variant 3	prostaglandin E receptor activity, ligand-dependent nuclear receptor activity, rhodopsin-like receptor activity	D86098, AY429108	N/A	* donor (all = GT); acceptor (c & h = AG, r = TG)
4	NM_020161*	-/-	2/3	73	150	382	MSTB2	MaLR[1]	hypothetical protein DKFZp547H025 (*DKFZp547H025*)	folic acid binding, reduced folate carrier activity	AK055355, AL359944, CR749679	DA513601	* donor (all = GT); acceptor (c & h = AG, r = GG)
5	NM_007072*	+/-	8/10	51(17)	414	217	LTR33	ERVL[1]	HERV-H LTR-associating 2 (*HHLA2*)	unknown	BC035971, AF126162, AK000692, AK027132	DB232201, BG283385	GT/AG
6	NM_001025468	+/+	2/3	95	97	408	MSTA	MaLR[1]	chromosome 3 open reading frame 47 (*C3orf47*)	undefined	BC093941, AK091470, BC101739	DA499220, BF514892, AL040547, BM709191	* GT/AG
7	NM_145027*	-/+	20/23	51(17)	814	361	MLT1A0	MaLR[1]	kinesin family member 6 (*KIF6*)	ATP binding, nucleotide binding, microtubule motor activity, microtubule-based movement	AL832634, BC022074, AK131471,	CA314029, BM696570, BI118088, BG189781, CV024391, BG715118	GT/AG

Supplementary Table 1 (continued)

8	NM_052962*	-/-	4/7*	96(32)	263	445	MSTB2	MaLR[1]	interleukin 22 receptor, alpha 2 (*IL22RA2*), transcript variant 1	interleukin-22 receptor activity, hematopoietin/ interferon-class (D200-domain) cytokine receptor activity	AY040567, AJ313162, AY358737	N/A	* donor (c & h = GT, r = AT); acceptor (all = AG)
9	NM_024728*	+/-	13/15	78(26)	434	519	MLT1E2	MaLR[1]	chromosome 7 open reading frame 10 (*C7orf10*)	transferase activity	AK021870, BC098318	BG678361, CN259576	GT/AG
10	NM_174930	+/+	5/6	89	134	387	MSTA	MaLR[1]	postmeiotic segregation increased 2-like 5 (*PMS2L5*)	ATP binding, damaged DNA binding (in mismatch repair process)	BC027480, D38436, D38501, D38502, D38439	BQ049786, DA486906, BE883788, AA621085, CA442493, AA411808, AA917605, AA282042	* donor (c & h = GT, r = AT); acceptor (all = AG)
11	NM_002679	-/-	7/8	89	297	387	MSTA	MaLR[1]	postmeiotic segregation increased 2-like 2 (*PMS2L2*)	unknown (in mismatch repair process)	AB017005	BM547367, BE703998, BE703901, BE701175, DR001682, AU120696, DR007875, BE163029	* donor (c & h = GT, r = AT); acceptor (all = AG)
12	NM_002679	-/-	2/8	89	297	387	MSTA	MaLR[1]	postmeiotic segregation increased 2-like 2 (*PMS2L2*)	unknown (in mismatch repair process)	AB017005	BM547367, BE703998, BE703901, BE701175, DR001682, DR007875, BE163029, BQ049786	* donor (c & h = GT, r = AT); acceptor (all = AG)
13	NM_002679	-/-	7/8	89	297	387	MSTA	MaLR[1]	postmeiotic segregation increased 2-like 2 (*PMS2L2*)	unknown (in mismatch repair process)	AB017005	BM547367, BE703998, BE703901, BE701175, DR001682, DR007875, BE163029, BQ049786	* donor (c & h = GT, r = AT); acceptor (all = AG)

Supplementary Table 1 (continued)

14	NM_002679	-/-	2/8	89	297	383	MSTA	MaLR[1]	postmeiotic segregation increased 2-like 2 (*PMS2L2*)	unknown (in mismatch repair process)	AB017005, AB017007	BE703998, BE703901, DR001682, AU120696, BG740816, DR007875, BE163029, BQ049786	* donor (c & h = GT, r = AT); acceptor (all = AG)
15	NM_032958*	-/-	3/8*	87(29)	116	384	MSTA	MaLR[1]	DNA directed RNA polymerase II polypeptide J-related gene (*POLR2J2*), transcript variant 2	DNA binding, transferase activity, protein dimerization activity, DNA-directed RNA polymerase activity (a subunit of RNA polymerase II)	BC056864, BC086857, BC071870, CR606303, CR624568, CR596358, CR614811, AJ277740	BM915750, BM559191, BG177266, DA093808, AL535064, DA239331, DA474156, AL556716	* donor (c & h = GT, r = AT); acceptor (all = AG)
16	NM_001023564*	+/-	4/5	94	218	419	MSTC	MaLR[1]	cathepsin L-like protein (*HCTSL-s*)	cysteine-type peptidase activity	AJ851862	N/A	no orthologous exonic sequence in chimpanzee
17	NM_017418*	+/-	6/8	74	70	371	MSTD	MaLR[1]	deleted in esophageal cancer 1 (*DEC1*)	unknown (a candidate tumor suppressor gene for esophageal squamous cell carcinomas located in a region commonly deleted in these carcinomas)	AB022761	N/A	GT/AG
18	NM_001010910*	+/-	2/3	85	102	203	MLT1J	MaLR[1]	hypothetical LOC399706 (*LOC399706*)	iron ion binding, electron transporter activity	AK097673	DB045405	no orthologous exonic sequence in rhesus monkey
19	NM_001548	+/+	2/3*	109	41	402	MLT1B	MaLR[1]	interferon-induced protein with tetratricopeptide repeats 1 (*IFIT1*), transcript variant 2	binding, immume response (interferon-induced protein)	AK092813	DB003897, DA380195, DA671459, DA672179, DA599173, DA676435, DA540285, DA599173	donor (c & h = GT, r = AT); acceptor (all = AG)
20	NM_015430*	-/+	7/12*	51(17)	737	348	MLT1A0	MaLR[1]	regeneration associated muscle protease (*DKFZP586H2123*), transcript variant 1	trypsin activity, peptidase activity, calcium ion binding, chymotrypsin activity	BC038457, BC089434, AK027841	DA475973, BG403264, DA543024, BX439313	GT/AG
21	NM_001001681*	+/+	3/7	104	129	395	MSTD	MaLR[1]	FLJ45300 protein (*FLJ45300*)	undefined	AK127233	DA313959	* GT/AG

Supplementary Table 1 (continued)

22	NM_173580*	+/-	2/3	107	122	133	MER21C	ERV1[1]	chromosome 11 open reading frame 44 (*C11orf44*)	undefined	AK096377	DA748198	no orthologous exonic sequence in rhesus monkey
23	NM_183378	-/+	26/28	107	1134	436	MER34B	ERV1[1]	ovochymase 1 (*OVCH1*)	peptidase activity,serine-type endopeptidase activity	BN000128	N/A	* GT/AG
24	NM_031915*	+/-	5/15	36(12)	719	50	MER34	ERV1[1]	SET domain, bifurcated 2 (*SETDB2*)	DNA binding, zinc ion binding, methyltransferase activity, histone-lysine N-methyltransferase activity (likely a histone H3 methyltransferase)	AF334407	N/A	GT/AG
25	NM_145019*	+/-	3/5	108(36)	582	675	LTR9	ERV1[1]	hypothetical protein FLJ30707 (*FLJ30707*)	structural constituent of ribosome	AK096364	DA745625	* donor (c & h & r = GT); acceptor (c & h = AG, r = deletion)
26	NM_001014830	-/+	3/5	75(25)	324	327	MLT1A0	MaLR[1]	hypothetical protein LOC196913 (*LOC196913*)	undefined	AF390030	DA935043, DA947999	GT/AG
27	NM_033141*	-/+	11/13	42(14)	1118	589	MLT2B3	ERVL[1]	mitogen-activated protein kinase kinase kinase 9 (*MAP3K9*)	ATP binding, nucleotide binding, transferase activity, MAP kinase kinase activity, protein-tyrosine kinase activity, JUN kinase kinase kinase activity, protein homodimerization activity, protein serine/ threonine kinase activity	AF251442, BX648924	N/A	GT/AG
28	NM_007319*	+/-	8/11*	92	374	1701	MER52A	ERV1[1]	presenilin 1 (Alzheimer disease 3) (*PSEN1*), transcript variant I-374	protein binding, Notch receptor processing, amyloid precursor protein catabolism (regulation amyloid precursor protein (APP) processing through their effects on a gamma-secretase, an enzyme that cleaves APP; involvement in the cleavage of the Notch receptor)	U40380, AF416717	N/A	no orthologous exonic sequences in chimpanzee and rhesus monkey
29	NM_020552*	+/-	2/5	67	105	129	MLT1D	MaLR[1]	T-cell leukemia/ lymphoma 6 (*TCL6*), transcript variant TCL6b1	unknown (a candidate gene for leukemogenesis)	AB035335	BX390485	donor (c & h = GT, r = GA); acceptor (all = AG)

Supplementary Table 1 (continued)

30	NM_020553*	+/-	5/8	67	119	129	MLT1D	MaLR[1]	T-cell leukemia/ lymphoma 6 (*TCL6*), transcript variant TCL6c1	unknown (a candidate gene for leukemogenesis)	AB035337	BX390485	donor (c & h = GT, r = GA); acceptor (all = AG)
31	NM_020554*	+/-	5/8	67	163	129	MLT1D	MaLR[1]	T-cell leukemia/ lymphoma 6 (*TCL6*), transcript variant TCL6d1	unknown (a candidate gene for leukemogenesis)	AB035338	BX390485	donor(c & h = GT, r = GA); acceptor (all = AG)
32	NM_005624*	+/-	4/5	120(40)	150	390	MLT1K	MaLR[1]	chemokine (C-C motif) ligand 25 (*CCL25*)	hormone activity, chemokine activity, chemotaxis, sensory perception, inflammatory response, G-protein coupled receptor protein signaling pathway	U86358	BX106823, AA295945	GT/AG
33	NM_005867*	-/-	2/3	102(34)	118	364	MLT2C1	ERVL[1]	Down syndrome critical region gene 4 (*DSCR4*)	unknown (contribution to the pathogenesis of many characteristics of Down syndrome, including morphological features, hypotonia, and mental retardation)	AB000099, BC069729, BC096162, BC096163, BC096164	CB993317, AW664531, BX112350, CD243838	GT/AG
34	NM_003159*	+/+	19/21*	84(28)	1030	398	MLT1J	MaLR[1]	cyclin-dependent kinase-like 5 (*CDKL5*), transcript variant I	ATP binding, nucleotide binding, transferase activity, protein-tyrosine kinase activity, protein serine/ threonine kinase activity, protein amino acid phosphorylation (association with X-linked infantile spasm syndrome (ISSX))	AY217744, Y15057, BC036091	N/A	no orthologous exonic sequence in chimpanzee
35	NM_024332*	+/-	8/12*	75(25)	316	528	MLT1J	MaLR[1]	chromosome X open reading frame 53 (*CXorf53*), transcript variant 1	unknown	BC002999, BC006540, AY438030, S68015	BU570969	no orthologous exonic sequences in chimpanzee and rhesus monkey
36	NM_207358*	-/-	4/10	118	139	1711	HERV16	ERVL[2]	hypothetical protein LOC339789 (*LOC339789*)	undefined	BC043563, AK127578	BI911310	donor (all = GT); acceptor (c & h = AG, r = AT)
37	NM_001004352*	+/+	4/7	187	141	5613	HERVL-A2	ERVL[2]	FLJ16323 protein (*FLJ16323*)	hydrolase activity, dUTP metabolism, nucleotide metabolism	AK131322	N/A	no orthologous exonic sequence in chimpanzee

Supplementary Table 1 (continued)

38	NM_004052*	-/+	3/6	85	194	87	HERV70	ERV1[2]	BCL2/adenovirus E1B 19kDa interacting protein 3 (*BNIP3*), nuclear gene encoding mitochondrial protein	induce apoptosis, anti-apoptosis, defense response to virus	BC009342, CR626676, BC021989, BC009342, AF002697, AK222626, BC067818, BC080643	CR995431, BX377664, BX404184, BX361486, BX361092, BX402244, BM799844, BM906527, BX445440, BM460721	GT/AG (GT position is not covered by HERV70)
39	NM_004052*	-/+	2/6	151	194	154	HERV70	ERV1[2]	BCL2/adenovirus E1B 19kDa interacting protein 3 (*BNIP3*), nuclear gene encoding mitochondrial protein	induce apoptosis, anti-apoptosis, defense response to virus	BC009342, CR626676, BC021989, BC009342, AF002697, AK222626, BC067818, BC080643	CR995431, BX377664, BX404184, BX361486, BX361092, BX402244, BM799844, BM906527, BM460721, BX333924	GT/AG (GT position is not covered by HERV70)
40	NM_014317*	+/+	2/12	33(11)	415	1493	THE1D-int	MaLR[2]	prenyl (decaprenyl) diphosphate synthase, subunit 1 (*PDSS1*)	transferase activity, isoprenoid biosynthesis	AK024802, AK223414, CR591586, AB210838, CR609440, BC063635, BC049211	DA565436, BX404180, CB996378, AL558274, CN297657, CN297656, AL560834, BF982221, DA969880, BI761095, BI916825, BQ212558, DR155397, CB152847	* GT/AG

Supplementary Table 1 (continued)

41	NM_080661*	+/+	2/7	124	333	701	MER57B-int	ERV1[2]	glycine-N-acyltransferase-like 1 (*GLYATL1*)	Acyltransferase activity, transferase acivity	BC008353, AK091965, AX747281	DA082180, T80698, DA355423, BM924769, DA631846, BE265837, CA397661, CF122488	no orthologous exonic sequence in rhesus monkey
42	NM_016488*	+/+	12/13*	225(75)	458	2904	HERVK22	ERVK[2]	periphilin 1 (*PPHLN1*), transcript variant 1	keratinization (may play a role in epithelial differentiation and contribute to epidermal integrity and barrier formation)	AY039238	N/A	* GT/AG
43	NM_015138*	+/+	14/18	58	585	85	HERVIP10F	ERV1[2]	Rtf1, Paf1/RNA polymerase II complex component, homolog (S. cerevisiae) (*RTF1*)	undefined	D87440, BC015052	CX783855, BQ421107, CN334996, DB063883, CB989235, BF795182, CN334990, AU127092, BQ230838, BQ354365	GT/AG (T position is not covered by HERVIP10F)
44	NM_015547*	+/+	16/17*	153	607	3404	MER9, HERVK9	ERVK[3]	acyl-CoA thioesterase 11 (*ACOT11*), transcript variant 1	acyl-CoA thioesterase activity, hydrolase activity, serine esterase activity	AB014607, AF416921	N/A	* GT/AG
45	NM_016488*	+/+	11/13*	101	458	1356	LTR22A, HERVK22	ERVK[3]	periphilin 1 (*PPHLN1*), transcript variant 1	keratinization (may play a role in epithelial differentiation and contribute to epidermal integrity and barrier formation)	AY039238, BC025306	BI258755, BM838224	* GT/AG
**first CDS exon**													
1	NM_144663*	-/-	1/4	141(47)	217	580	LTR18B	ERVL[1]	chromosome 11 open reading frame 40 (*C11orf40*)	undefined	AF439154	N/A	* donor (c & h = GT, r = GA), no flanking acceptor: first exon of gene

Supplementary Table 1 (continued)

2	NM_001012274*	+/+	2/3	106	117	998	MER61A, MER61A-int	ERV1[3]	chromosome 1 open reading frame 99 (*C1orf99*)	undefined	BC040856	BM452121	* GT/AG
**last CDS exon**													
1	NM_013329*	-/-	16/16*	114(38)	815	631	MER39B	ERV1[1]	chromosome 21 open reading frame 66 (*C21orf66*), transcript variant 2	DNA binding, regulation of DNA-dependent transcription	AY033904	N/A	* acceptor (c & h & r = AG), no flanking donor: last exon of gene
2	NM_015845*	-/+	14/14*	190	586	656	MLT2F	ERVL[1]	methyl-CpG binding domain protein 1 (*MBD1*), transcript variant 2	metal ion binding, methyl-CpG binding, transcription corepressor activity	AF078831	N/A	acceptor(c & h & r = AG), no flanking donor: last exon of gene
3	NM_181538*	-/-	2/2	59	279	491	LTR72B	ERV1[1]	gap junction protein, epsilon 1, 29kDa (*GJE1*)	connexon channel activity	AF503615, AY297109	N/A	* acceptor (c & h & r = AG), no flanking donor: last exon of gene
4	NM_001015884	-/-	4/4	33(11)	115	384	MSTA	MaLR[1]	RPB11b2alpha protein (*POLR2J3*)	undefined	AJ277741	BI223488, BE936699, DW433233	* acceptor (c & h & r = AG), no flanking donor: last exon of gene
**single protein coding exon**													
1	NM_001007236	-/-	1/1	5640 (1880)	1879	7536	HERVK	ERVK[2]	endogenous retroviral sequence K, 6 (*ERVK6*)	aspartic-type endopeptidase activity, metal ion binding, nucleic acid binding, peptidase activity, zinc ion binding, DNA transposition, proteolysis, virus life cycle	Partial CDS: AF080233, DQ069911, DQ069913, U87590, U87591, U87592	N/A	no orthologous exonic sequence in chimpanzee and rhesus monkey
2	NM_001007236 (different genomic location from the previous entry)	-/-	1/1	5640 (1880)	1879	7535	HERVK	ERVK[2]	endogenous retroviral sequence K, 6 (*ERVK6*)	aspartic-type endopeptidase activity, metal ion binding, nucleic acid binding, peptidase activity, zinc ion binding, DNA transposition, proteolysis, virus life cycle	Partial CDS: AF080233, DQ069911, DQ069913, U87590, U87591, U87592	N/A	no orthologous exonic sequence in chimpanzee and rhesus monkey
3	NM_001007253	-/-	1/1	1696	564	8428	HERV3	ERV1[2]	endogenous retroviral sequence 3 (includes zinc finger protein H-plk/HPF9) (*ERV3*)	unknown	N/A	N/A	no orthologous exonic sequence in chimpanzee

(1) RefSeq gene accession number. The entries marked with arterisk are known protein coding genes with the products listed in SWISS-PROT, TrEMBL, and TrEMBL-NEW and their corresponding mRNAs presented in the GenBank records. The adjacent entries in gray shading indicate the redundant exons, exons with exact or overlap boundaries participated in different alternative transcripts (derived from the same LRTS). Item 10-14 of internal protein coding exons could be the result of duplication of genes or gene regions, rather than direct LTR element insertion.
(2) Orientation of gene and LRTS assigned as gene strand/ LRTS strand. +, sense strand; -, antisense strand
(3) The position of exon on a gene/ total number of exons. The arterisk indicates the alternatively spliced exon (exons which are not present in all transcript variants).
(4) The length of LRTS-derived exon. In case of length divisible by three, the number in parenthesis shows the length of additional amino acid sequence provided by LRTS.
(5) The length of LRTS fragment covering the exon.
(6) Class/ family of LRTS. The superscript number refers to the part of LRTS covering an exon. 1, only LTR; 2, only internal sequence (sequence between flanking LTRs containing traditional viral genes); 3, both LTR and internal sequence.
(7) Gene descriptions according to the Gene Ontology (GO). undefined: no match for the gene in GO. unknown: gene product whose process, function, or localization is not known or cannot be inferred.
(8) GenBank mRNAs and (9) ESTs confirming the existence of LRTS-derived exon in human. Data were derived from the UCSC genome browser and the Entrez Gene.
(10) Sequences in place of splice sites flanking the LRTS-derived exon compared between human (May 2004, hg17), chimpanzee (Nov 2003, panTro1) and rhesus monkey (Jan 2006, rheMac2). h, human; c, chimpanzee; r, rhesus monkey. The data were derived from the multiple sequence alignment of the target exon of human and homologous sequences of chimpanzee and rhesus monkey retrieved from the UCSC genome browser. The entries marked with arterisk suggest possible primate-specific LTR element insertion (the homologous LRTS regions are present in chimpanzee and rhesus monkey but not in other vertebrates; mouse, rat, rabbit, dog, cow, armadillo, elephant, tenrec, opossum, chicken, frog, zebrafish, tetraodon, fugu).
